# Expression of Signal Transduction System Encoding Genes of *Yersinia pseudotuberculosis* IP32953 at 28°C and 3°C

**DOI:** 10.1371/journal.pone.0025063

**Published:** 2011-09-20

**Authors:** Eveliina Palonen, Miia Lindström, Reija Karttunen, Panu Somervuo, Hannu Korkeala

**Affiliations:** Department of Food Hygiene and Environmental Health, Faculty of Veterinary Medicine, University of Helsinki, Helsinki, Finland; Universität Münster, Germany

## Abstract

*Yersinia pseudotuberculosis* is a significant psychrotrophic food pathogen whose cold tolerance mechanisms are poorly understood. Signal transduction systems serve to monitor the environment, but no systematic investigation of their role at cold temperatures in *Y. pseudotuberculosis* has yet been undertaken. The relative expression levels of 54 genes predicted to encode proteins belonging to signal transduction systems in *Y. pseudotuberculosis* IP32953 were determined at 28°C and 3°C by quantitative real-time reverse transcription-PCR. The relative expression levels of 44 genes were significantly (p<0.05) higher at 3°C than at 28°C. Genes encoding the two-component system CheA/CheY had the highest relative expression levels at 3°C. Mutational analysis revealed that *cheA* is important for growth and motility at 3°C. The relative expression level of one gene, *rssB*, encoding an RpoS regulator, was significantly (p<0.05) lower at 3°C than at 28°C. The results suggest that several signal transduction systems might be used during growth at low temperature, and at least, CheA/CheY two-component system is important for low-temperature growth.

## Introduction

*Yersinia pseudotuberculosis* is an important food-borne pathogen capable of growing at refrigeration temperatures and under modified atmospheres [1]. Typical symptoms of yersiniosis resulting from mesenteric lymphadenitis of the small intestine, such as fever and acute abdominal pain [2], are often mistaken for appendicitis and have led to unnecessary appendectomies [3]. Apart from infections due to contaminated drinking water [4], [5], *Y. pseudotuberculosis* has caused outbreaks through contaminated fresh produce stored at low temperature [6]–[8]. While low temperature efficiently controls the growth of many pathogenic bacteria, it readily favors the growth of *Y. pseudotuberculosis* [9]. Although, little is known about the cold tolerance mechanisms of *Y. pseudotuberculosis* (reviewed in [1]), key changes reported to occur in *Enterobacteriaceae* during adaptation to growth at cold temperatures include an increase in low-melting-point lipids in cell membranes and accumulation of compatible solutes in the cells [1].

To survive, bacteria must sense changes in temperature and other extrinsic circumstances. Two-component signal transduction systems are widespread among bacteria and help them monitor and adapt to changes in their extra- or intracellular environment [10]. A classical two-component system consists of a sensor histidine kinase and a response regulator located in the cell membrane and cytoplasm, respectively [10]. In response to a specific stimulus, histidine kinase autophosphorylates. Subsequently, the phosphoryl group is transferred to the response regulator, which activates and binds to the DNA, resulting in changes in transcription [10]. A variant of the classical two-component system is a multistep phosphorelay consisting of a hybrid histidine kinase, a histidine phosphotransferase, and a response regulator [10]. In response to a stimulus, hydrid histidine kinase autophosphorylates. This is then followed by intramolecular transmission of the phosphoryl group to the response regulator-like receiver domain of the hydrid histidine kinase. The histidine phosphotransferase further transfers the phosphoryl group to the response regulator [10]. The number of two-component systems varies between bacteria [11]. While *Mycoplasma genitalium* has none, *Synechocystis sp.* has 80 two-component proteins [11]. Predicted by *in silico* analysis, the *Y. pseudotuberculosis* strain IP32953 has 24 complete signal transduction systems and 5 orphan hybrid histidine kinases or response regulators [12].

Several studies have investigated the function of some signal transduction systems in *Y. pseudotuberculosis*. The PhoQ/PhoP [13]–[17], CpxA/CpxR [18], [19], and BarA/UvrY [20] two-component systems as well as the CvgSY [21] hybrid histidine kinase play a role in virulence in *Y. pseudotuberculosis*. The Rcs multistep phosphorelay affects stress survival and virulence [22], and the response regulator OmpR is involved in acid tolerance and flagella biosynthesis [23], [24]. The YfhK/YfhA two-component system is involved in the amino sugar metabolism [25], while PmrB/PmrA may be related to peptidoglycan homeostasis [14]. In addition, one study used gene inactivation to investigate the role of the 24 predicted response regulators in *Y. pseudotuberculosis* in resistance to conditions faced in the gastrointestinal tract of the host [26]. The mutants were screened for susceptibility to inorganic and organic acids, high salinity, polymyxin B, hydrogen peroxide, and sodium choleate. Tolerance to one type of stress was altered in four mutants (*rcsB*, *ntrC*, *rstA*, and *yfhA*) and to several types of stresses in four mutants (*ompR*, *arcA*, *phoP*, and *pmrA*). Furthermore, *ompR*, *phoP*, *rstA*, and *yfhA* were shown to play roles in virulence [26]. The roles of the signal transduction systems of *Y. pseudotuberculosis* at low temperature remain unknown.

As refrigeration is the most important preservation method used in the modern food industry, adaptation to low temperature is a key for bacteria to survive in the food chain. *Y. pseudotuberculosis* tolerates cold well and has an advantage over most mesophilic bacteria in foods. However, the mechanisms underlying the cold tolerance of *Y. pseudotuberculosis* remain poorly understood. A systematic investigation of all the signal transduction systems of *Y. pseudotuberculosis* at low temperature would provide information on how these key sensory and regulatory elements are involved in cold tolerance. The aim of this study was to monitor the expression of all the predicted signal transduction system encoding genes in *Y. pseudotuberculosis* IP32953 at 3°C relative to their expression at 28°C using quantitative real-time reverse transcription-PCR (RT-qPCR). Several signal transduction systems seemed to be involved in adaptation to cold temperature. Results were confirmed by selective mutants and their phenotypical characterization.

## Materials and Methods

### Bacterial strain and growth conditions

*Y. pseudotuberculosis* strain IP32953 is a clinical isolate from a human patient and completely sequenced [27]. The strain was gratefully received from Dr. Elisabeth Carniel (Institut Pasteur, Paris, France). *Y. pseudotuberculosis* was grown in Luria-Bertani (LB) broth with shaking or on LB agar plates (BD, Franklin Lakes, New Jersey, USA) at 28°C or 3°C representing the optimum growth temperature [28] and a stressful temperature allowing detectable growth, respectively. *Escherichia coli* strain comparable to DH5α (Sigma-Aldrich Co., St. Louis, Missouri, USA) was grown at 37°C with shaking in LB broth or on LB agar (BD) supplemented with 1% glucose (Sigma-Aldrich Co.), 100 µg/ml ampicillin (Sigma-Aldrich Co.), 25 µg/ml chloramphenicol (Sigma-Aldrich Co.) or 50 µg/ml kanamycin (Sigma-Aldrich Co.) when appropriate.

### RNA isolation

**Figure 1:**
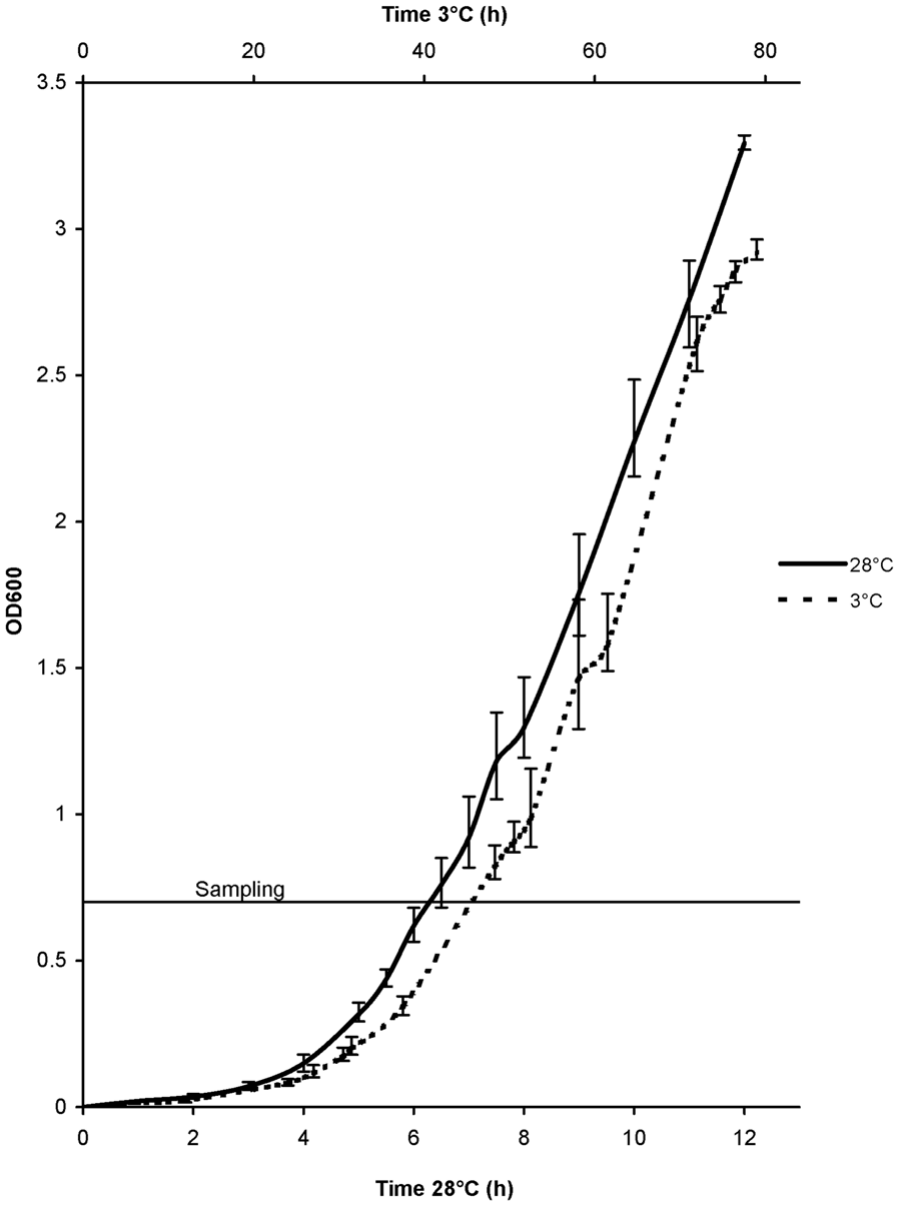
Growth curves of *Yersinia pseudotuberculosis* IP32953 at 3°C and 28°C.

*Y. pseudotuberculosis* IP32953 was grown at 28°C on LB agar for 24 h. Three colonies (biological replicates) were inoculated and grown separately in LB broth overnight, inoculated into fresh LB (1∶100), and grown at 28°C or 3°C to an early logarithmic growth phase (OD_600_ of 0.7, corresponding to 10^8^ viable cells/ml, Figure 1). Samples for total RNA extraction were collected by mixing 2×10 ml of bacterial culture with a cold phenol-ethanol mixture (1∶9) and kept on ice for 30 min. Samples were subsequently centrifuged at 2°C at 5000× *g* for 15 min, and the resulting cell pellets were stored at −70°C until RNA isolation. The total RNA was isolated using an RNeasy Midi kit (Qiagen GmbH, Hilden, Germany) with on-column DNase digestion using an RNase-free DNase set (Qiagen GmbH) according to the manufacturer's instructions. An additional DNase treatment was performed with a DNA-free kit (Applied Biosystems, Foster City, California, USA) according to the manufacturer's instructions. RNA concentration and quality were measured with a Nanodrop ND-1000 spectrophotometer (Thermo Fisher Scientific Inc., Waltham, Massachusetts, USA). An A_260_/A_280_ ratio of 2.0 (varied between 2.06–2.13) was considered pure RNA. RNA integrity was examined using an Agilent 2100 Bioanalyzer (Agilent Technologies Inc., Santa Clara, California, USA). RNA was stored at −70°C until use.

### Reverse transcription

Each RNA sample was reverse transcribed into cDNA in duplicate (RT replicates) using a Dynamo cDNA Synthesis kit (Finnzymes Oy, Espoo, Finland) according to the manufacturer's instructions. RNA was incubated for five min at 65°C to destabilize secondary structures. The total reaction volume for reverse transcription was 20 µl and contained 300 ng of random hexamers, 900 ng of RNA, and 2 µl of M-MuLV RNase H+ reverse transcriptase. The reactions were incubated at 25°C for 10 min, at 37°C for 30 min, and at 85°C for 5 min. To control DNA contamination, minus-RT controls were prepared from RNA samples by adding all the reaction components except the reverse transcriptase. The cDNAs were stored at −20°C before use in RT-qPCR.

### RT-qPCR

RT-qPCR was performed using a Dynamo Flash SYBR Green qPCR kit (Finnzymes Oy) according to the manufacturer's instructions. Primers were designed for the predicted signal transduction system genes of IP32953 [12], [29], the reference gene 16S rRNA gene (Table 1), and the *ibpA* encoding a heat shock protein (GenBank accession number BX936398) by using Primer3 software [30] (http://frodo.wi.mit.edu/primer3/) (Table S1). The total reaction volume was 20 µl, including 4 µl of template cDNA and 0.5 µM of each primer. PCR runs were performed with a Rotor-Gene 3000 Real Time Thermal Cycler (Qiagen GmbH). The amplification protocol consisted of initial denaturation at 95°C for 7 min, 40 cycles of denaturation at 95°C for 10 s, annealing at 60°C for 15 s, extension at 72°C for 20 s, and a final extension at 60°C for 1 min. Fluorescence data were acquired at the end of each extension step. After each run, a melt curve analysis was performed by raising the temperature at a rate of 0.5°C/5 s from 60°C to 98°C to confirm specificity. Minus-RT controls yielded no specific products, thus indicating no DNA contamination. Dilution series of pooled cDNA originating from the RT replicates of the biological replicates at 28°C and 3°C were amplified in triplicate (PCR replicates) to determine standard curves and thus the amplification reaction efficiencies for each primer pair. The reaction efficiency, based on the dilution series described above, was determined for 16S rRNA gene for four dilution series, and for the genes encoding signal transduction systems and *ibpA* for the dilution series from the cDNAs to be used in subsequent runs of the gene. For each primer pair, threshold fluorescence levels were set automatically with Rotor-Gene 3000 software, and reaction efficiencies were calculated as 10^(−1/M)^-1, where M is the slope of the straight line from a semilogarithmic plot of the quantification cycle (C_q_) as a function of the cDNA concentration using Rotor-Gene 3000 software. Reaction efficiencies for 16S rRNA gene and signal transduction system encoding genes appear in Table S2 and Table S3, reaction efficiency of *ibpA* was 0.92.

**Table 1:** Verification of the use of 16S rRNA gene as a reference gene at low temperature in *Yersinia pseudotuberculosis* IP32953.

Parameter between 3°C and 28°C	Value
Arithmetic mean (C_q_)	15.82
Geometric mean (C_q_)	15.81
Average deviation	0.47
CV(% C_q_)^a^	2.96

a CV, coefficient of variance as a percentage of the C_q_ value.

For PCR amplification, the cDNA was diluted to 1∶20 for amplification of the genes that encode signal transduction systems and *ibpA*, and 1∶100000 for the amplification of 16S rRNA gene. Each cDNA sample was analyzed in duplicate (PCR replicates). The threshold levels acquired from the standard curves served in the analysis. The duplicate C_q_ values for the PCR replicates were averaged. With the average C_q_s, the relative expression levels of the genes that encode signal transduction systems and *ibpA* at 3°C, normalized to 16S rRNA gene and calibrated to samples taken at 28°C, were quantified for each RT replicate by calculating the expression ratios (R) with equation 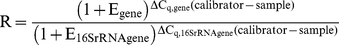 [31], where E_gene_ is the amplification reaction efficiency of a signal transduction system gene or *ibpA* transcript, E_16S rRNA gene_ is the amplification reaction efficiency of 16S rRNA gene transcripts, ΔC_q,gene_ is the C_q_ deviation between calibrator and sample for the signal transduction system gene or *ibpA* transcript, and ΔC_q,16S rRNA gene_ is the C_q_ deviation between calibrator and sample for the 16S rRNA gene transcripts. The resulting Rs were averaged for RT replicates. The student's t-test (Microsoft Excel) was performed for the biological replicates to test the differences between the relative expression levels of genes that encode signal transduction systems and *ibpA* at 3°C and 28°C.

### Mutagenesis

A *cheA*30–31::Ltr Kan^R^ mutant (hereafter called *cheA30*) was created using the TargeTron Gene Knockout System (Sigma-Aldrich Co.) following manufacturer's instructions. All the primers used in constructing and confirming mutants are listed in Table S4. Briefly, re-targeting PCR was performed with primers *cheA30*-IBS, *cheA30*-EBS1d, *cheA30*-EBS2 and EBS Universal. Resulting PCR product was digested and ligated into plasmid pACD4K-C, and transformed into *E. coli* by heat shock. The re-targeted plasmid clone was isolated by using a GeneJET Plasmid Miniprep Kit (Fermentas International Inc., Burlington, Ontario, Canada) and sequenced to confirm correct sequence with T7 primer. Electrocompentent *Y. pseudotuberculosis* IP32953 was made as described previously [32] and plasmid pAR1219 (Sigma-Aldrich Co.) was transformed into *Y. pseudotuberculosis* IP32953 using 0.1 cm cuvettes with 25 µF, 200 Ω and 1.8 kV. Subsequently, cells were incubated in super-optimal broth with catabolite repression (SOC) (Sigma-Aldrich Co.) for 3 hours and plated on LB agar with ampicillin. Electrocompentent *Y. pseudotuberculosis* IP32953 containing pAR1219 was made as described above and the re-targeted pACD4K-C was introduced into the cells. After 3-hour SOC incubation, cells were plated on LB agar with ampicillin and chloramphenicol. After 24 hours, colonies from the LB agar plate were inoculated and grown overnight in LB broth with ampicillin and chloramphenicol. Cells were inoculated into fresh LB broth (1∶50) with ampicillin and chloramphenicol and grown to an OD600 of 0.2. Expression and insertion of the intron were induced by adding 0.5 mM isopropyl β-D-thiogalactoside (IPTG) (Sigma-Aldrich Co.) and incubation was continued overnight. Cells were centrifuged and resuspended into fresh LB. After 3 hours incubation, cells were plated on LB agars containing kanamycin. Knockouts were confirmed by PCR using *cheA30*-flank-left and *cheA30*-flank-right, and *cheA30*-flank-left and EBS Universal primers. A *cheA243–244*::Ltr Kan^R^ mutant (*cheA243*) and a *cheY243–244*::Ltr Kan^R^ mutant (*cheY243*) were created similarly with primers *cheA243*-IBS, *cheA243*-EBS1d, *cheA243*-EBS2, EBS Universal; and *cheY243*-IBS, *cheY243*-EBS1d, *cheY243*-EBS2, EBS Universal, respectively. The *cheA243* was confirmed with primers *cheA243*-flank-left and *cheA243*-flank-right, and *cheA243*-flank-right and EBS Universal. The *cheY243* was confirmed with primers *cheY243*-flank-left and *cheY243*-flank-right, and *cheY243*-flank-left and EBS Universal. Primers N*inv*-left and N*inv*-right [33], and K*virF*-left and K*virF*-right [34] were used in PCR to confirm species *Y. pseudotuberculosis* and the presence of the virulence plasmid pYV, respectively. All mutants were cured of pAR1219 by several subcultures in LB broth without ampicillin. All the ampicillin-sensitive clones were ascertained to contain the insertion mutation by PCR with flank primers. Primers N*inv*-left and N*inv*-right [33], and K*virF*-left and K*virF*-right [34] were used in PCR to confirm mutants for *Y. pseudotuberculosis* with pYV, respectively.

### Southern blotting

Southern blotting was performed to confirm single intron insertion in the mutants. A PCR DIG Probe Synthesis Kit (Roche Applied Science, Penzberg, Germany) was used following manufacturer's instructions with primers probe-left and probe-right (Table S4) to synthesize a 199-bp digoxigenin-labelled probe. Pitcher's method [35] was used to extract genomic DNA from the wild type and from the mutants with the following changes. Tris-EDTA (10∶1) containing 0.4% sodium dodecyl sulphate, 220 µg/ml proteinase K and 2 mg/ml RNAse, was used for cell lysis. After adding ammonium acetate and incubating for 10 minutes on ice, 350 µl of phenol-chloroform-isoamyl alcohol (25∶24∶1) was added, samples were shaken rigorously, centrifuged, and Pitcher's method was continued with the supernatant. Genomic DNA from the wild type, *cheA30* and *cheA243* was digested with *Hind*III (New England Biolabs Inc., Ipswich, Massachusetts, USA), and from *cheY243* with *Xba*I (New England Biolabs Inc.). Digested DNAs and pACD4K-C (Sigma-Aldrich Co.) were shifted to a positively charged nylon membrane, hybridized with the probe and detected as recommended by Roche Applied Science.

### Growth experiments

Three separate colonies of the *Y. pseudotuberculosis* IP32953 wild type strain, *cheA30*, *cheA243*, and *cheY243* ampicillin sensitive mutant strains grown on LB agar, were separately inoculated into fresh LB broth and grown overnight with shaking. Subsequently, 1∶100 dilutions into fresh LB broth were accomplished and 300 µl of the dilutions were pipetted into wells of microtiter plates in triplicate. The microtiter plate was incubated at 3°C in the turbidity reader Bioscreen C MBR (Oy Growth Curves Ab, Helsinki, Finland). Turbidity of the cultures was measured at one-hour intervals after shaking for 20 s. Growth curves were acquired by plotting the ensuing OD_600_ values against time. Growth experiments were done likewise at 28°C but turbidity was measured at 20-minute intervals. Colony counting was performed to check that the overnight cultures contained similar amounts of viable cells. The correspondence of OD_600_ values to the amount of viable cells was ensured by performing bacterial colony counting using plate count agar (BD) from the wild type strain and from all the mutants.

### Motility tests

Motility test medium M103 with 2,3,5-triphenyl tetrazolium chloride [36] was modified to contain 0.3% agar. The IP32953 wild type strain and all the ampicillin sensitive mutants were stab-inoculated into M103 containing tubes. Tubes were incubated at 3°C, 22°C, 28°C and 37°C, and growth was monitored for 22 days, 9 days, 6 days and 6 days, respectively. Photographs were taken after 9 days at 3°C, 4 days at 22°C, 24 hours at 28°C and 24 hours at 37°C.

## Results

### Expression of signal transduction system encoding genes

The relative expression levels of 44 genes out of 54 genes were significantly higher (p<0.05) at 3°C than at 28°C (Table S3, in the order of expression ratios). The relative expression levels of *cheA* and *cheY*, which encode a CheA/CheY two-component system involved in chemotaxis, were 31- and 25-fold higher, respectively, at 3°C than at 28°C. 16S rRNA gene was used as a normalization reference for RT-qPCR. Its expression was stable between 3°C and 28°C (Table 1).

In 17 of the 24 predicted complete signal transduction systems (23 two-component systems and one multistep phosphorelay), both or all of the genes that encode a system had significantly higher relative expression levels at 3°C than at 28°C (Table S3). In 4 of the 24 complete signal transduction systems, the histidine kinase gene alone, and in two systems, the response regulator gene alone had significantly higher relative expression at 3°C than at 28°C. Of the 5 orphan signal transduction components, three response regulator genes had significantly higher relative expression levels at 3°C than at 28°C, and one of them, *YPTB1603*, had the third highest relative expression level at 3°C, 13-fold, compared to the expression level at 28°C (Table S3). The relative expression level of one gene, *YPTB2099*, was significantly lower at 3°C than at 28°C (Table S3). The UhpB/UhpA two-component system was the only one of the 24 for which the relative expression of neither the histidine kinase encoding gene *uhpB* nor the response regulator encoding gene *uhpA* differed with temperature (Table S3). Relative expression ratio of the heat shock protein encoding *ibpA* was 0.09 (p-value 0.05) indicating an 11-fold higher expression level at 28°C than at 3°C.

### Construction of mutants

Three different insertional knockout mutants were constructed for *cheA* and *cheY*. Group II intron was inserted in *cheA* either in sense orientation at position 30–31 or in antisense orientation at position 243–244. The *cheY* insertional mutant had the intron located at position 243–244 in sense orientation. Southern blotting analysis confirmed that all the mutants had a single intron inserted in the genome.

### Phenotypic characteristics of the mutants

**Figure 2:**
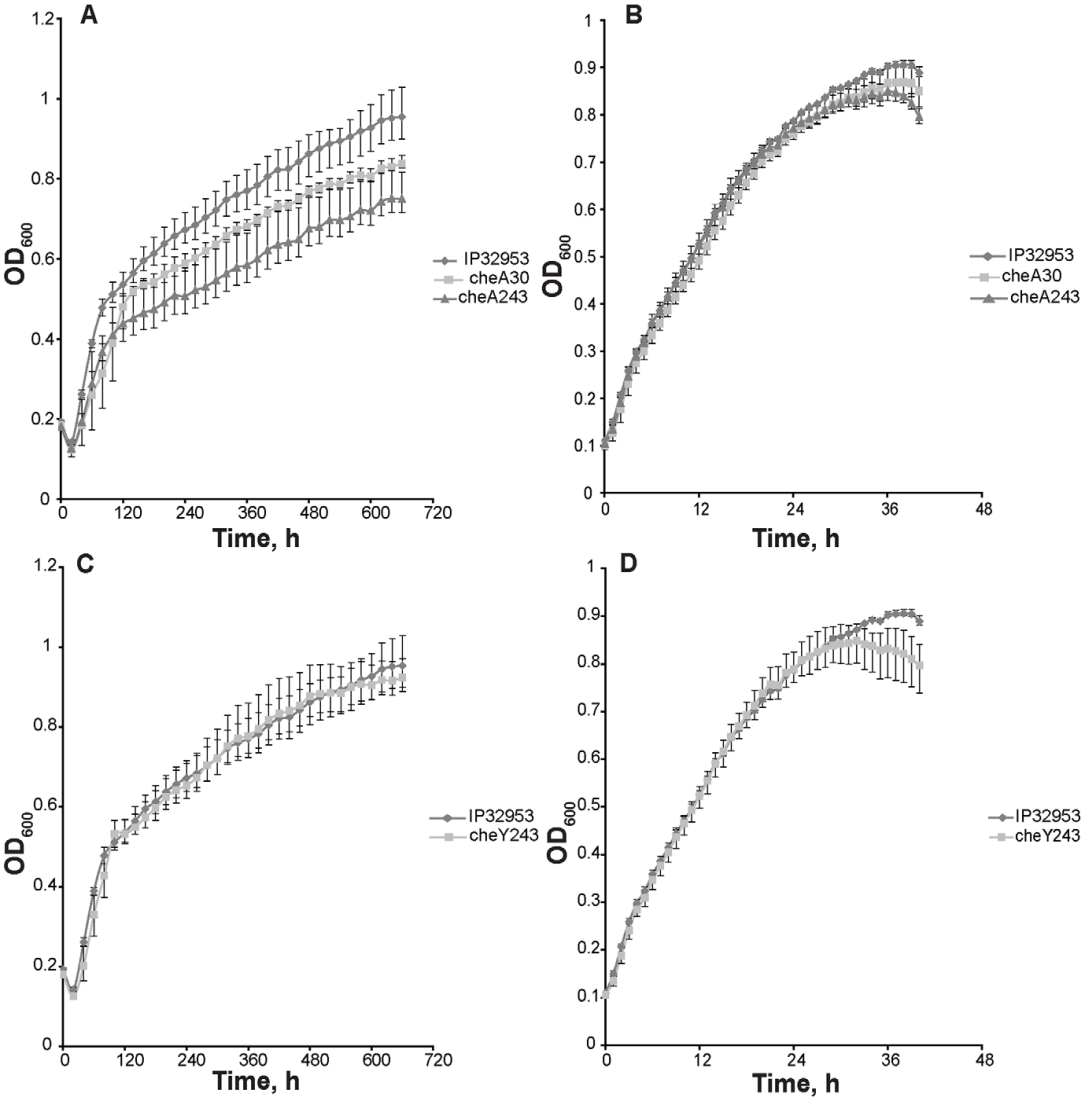
Growth curves of *Yersinia pseudotuberculosis* IP32953 wild type strain, *cheA*, and *cheY* mutants. Growth of the *cheA* mutants at 3°C (A) and 28°C (B), and the *cheY* mutant at 3°C (C) and 28°C (D). In graphs (A) and (C), measured values are shown in twenty-hour intervals, in (B) and (D), in one-hour intervals. Error bars represent minimum and maximum values.

Amount of 3×10^6^ viable cells of the wild type strain and the mutants were used in the growth experiments. The mutant *cheA243* had clearly impaired, and mutant *cheA30* had slightly impaired growth at 3°C (Figure 2A). The growth of the mutant *cheY243* did not differ from that of the wild type at 3°C (Figure 2C). Mutations did not affect growth at 28°C (Figures 2B and D). Colony counting of bacteria confirmed the correspondence of OD_600_ values to the number of viable cells.

**Figure 3:**
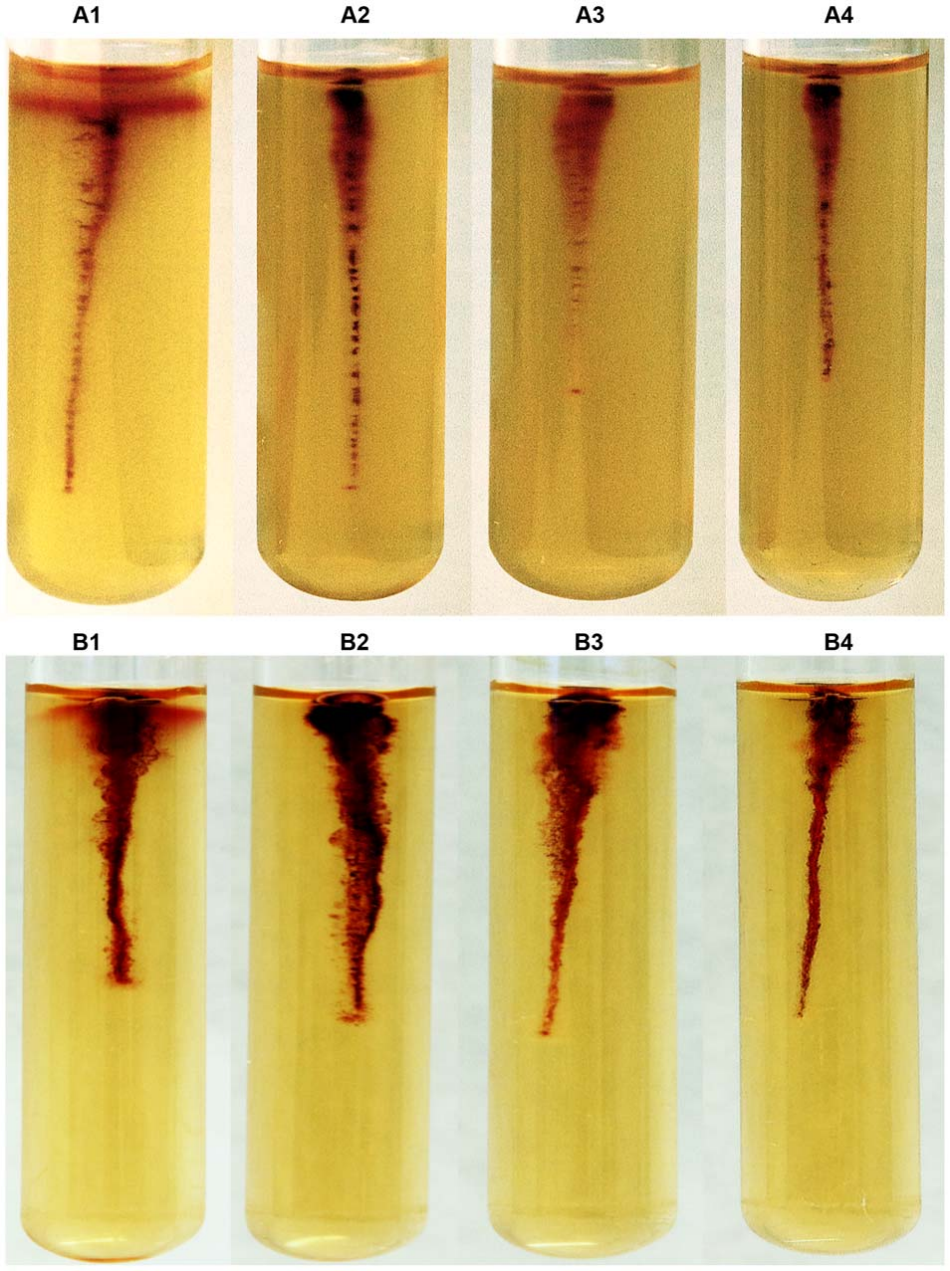
Motility tests of *Yersinia pseudotuberculosis* IP32953 wild type strain, and *cheA* and *cheY* mutants. (A) 3°C and (B) 22°C. IP32953 (1), *cheA30* (2), *cheA243* (3), and *cheY243* (4).

**Figure 4:**
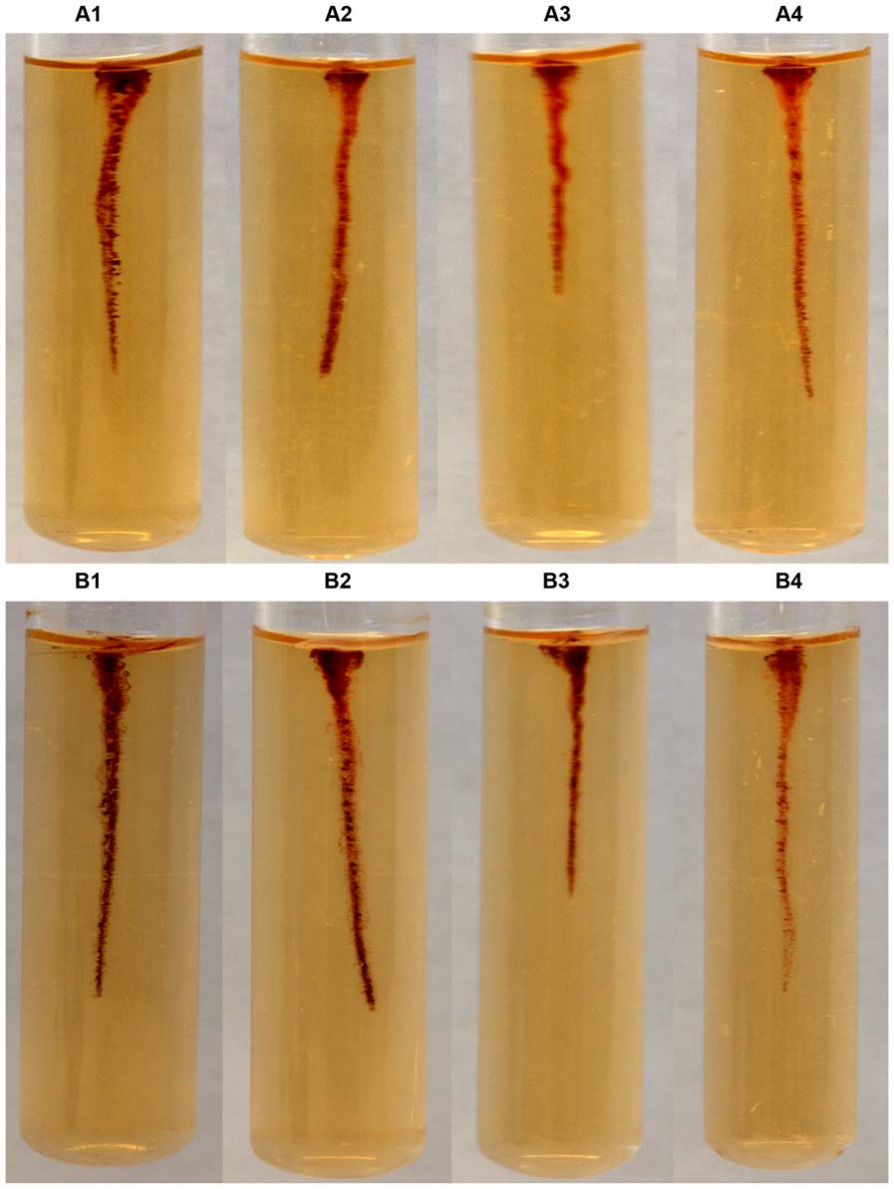
Motility tests of *Yersinia pseudotuberculosis* IP32953 wild type strain, and *cheA* and *cheY* mutants. (A) 28°C and (B) 37°C. IP32953 (1), *cheA30* (2), *cheA243* (3), and *cheY243* (4).

Motility tests were performed for the *cheA30*, *cheA243* and *cheY243* mutants, and for the wild type strain IP32953. At 3°C and 22°C, the wild type IP32953 showed umbrella type motility, while none of the *che* mutants was motile (Figures 3A and B). At 28°C or 37°C none of the strains was motile (Figures 4A and B).

## Discussion

The mechanisms *Y. pseudotuberculosis* uses to adapt to and to grow at low temperatures are of special interest due to the food safety risks this psychrotrophic pathogen poses to modern chilled foods. Bacteria use signal transduction systems to monitor the environment and to adjust their gene expression to changing conditions. *Y. pseudotuberculosis* strain IP32953 has 24 complete signal transduction systems and 5 orphan hybrid histidine kinases or response regulators [12], for which roles in virulence and stress response have been demonstrated (Table S5). The role of signal transduction systems in the growth of *Y. pseudotuberculosis* at low temperature is poorly understood. To better understand their involvement, we determined the relative expression levels of 54 genes predicted to encode signal transduction systems in *Y. pseudotuberculosis* IP32953 at 3°C and 28°C. The relative expression levels of the majority of signal transduction system encoding genes were significantly higher at 3°C than at 28°C (Table S3). This is in line with previous transcriptomic studies with DNA microarrays on the psychrotrophic *Listeria monocytogenes* and on the mesophilic *E. coli*, the latter belonging to the same family as *Yersinia*, that showed approximately one third of the signal transduction system encoding genes to be induced at low temperature [37]–[39]. The even greater proportion of up-regulated signal transduction genes in *Y. pseudotuberculosis* was somewhat surprising. Therefore the expression analysis of *ibpA*, encoding a heat shock protein and shown to be expressed at five-fold higher level at 30°C than at 10°C [40], was included in our study as a control. The observed 11-fold higher expression level at 28°C than at 3°C confirmed that our RT-qPCR detects both up-regulated and down-regulated genes and thus verifies the findings on the signal transduction genes. Moreover, the 16S rRNA gene was shown to be expressed at stable levels at both temperatures (Table 1), which indicates 16S rRNA gene is a suitable reference gene for cold stress studies in *Y. pseudotuberculosis*. It has also been shown to be the most stable housekeeping gene to be used in cold stress studies with *L. monocytogenes* [41].

The substantially higher expression levels of *cheA* and *cheY* (Table S3), encoding the two-component system CheA/CheY, at low temperature than at optimal growth temperature suggest that this two-component system plays a role in growth at low temperature. In a study conducted with a *Y. enterocolitica* transposon mutant library, *cheA* was also one of the most highly expressed genes during the early and mid-exponential growth phase at 10°C in relation to the expressions at 30°C [40]. Moreover, *cheY* expression in the mid-exponential growth phase at 4°C, compared to its expression at 37°C, was slightly induced, and *cheA* expression remained nearly stable when investigated with DNA microarrays in *Y. enterocolitica* [42]. The role of the CheA/CheY two-component system at low temperature is further supported by induced *cheY* expression after cold shock from 37°C to 15°C in *E. coli* [43].

Involvement of the CheA/CheY two-component system in *Y. pseudotuberculosis* during cold stress was further investigated by constructing insertional knockout mutants *cheA30*, *cheA243* and *cheY243*. Mutation in *cheA* resulted in impaired growth at 3°C (Figure 2A), which confirms the important role of the CheA sensor during growth at low temperature. The mutation in *cheY* did not affect the growth at 3°C (Figures 2C), which may suggest that CheY is not essential during growth at low temperature, or other regulators may compensate for it. It is also possible that, as the only successful mutation site (between nucleotides 243–244) was near the C-terminus of the 390-bp long *cheY*, a functional truncated N-terminus of CheY was sufficient to exert the growth of the *cheY* mutant at 3°C. At 28°C, the growth of none of the mutants differed from the wild type strain (Figures 2B and D).

Components of the chemotaxis signal transduction system are highly conserved and, in *E. coli*, consist of chemoreceptors and six Che proteins: CheA, CheW, CheY, CheZ, CheB, and CheR [44]. The transmembrane chemoreceptors monitor the environment, and together with the histidine kinase CheA and another protein CheW form receptor-signaling complexes that regulate the autophosphorylation of CheA and thus the phosphorylation of the response regulator CheY [44]. Phosphorylated CheY diffuses to flagellar motors where it promotes clockwise rotation of the flagella, leading to a tumbling motion that keeps the cell in place [44]. When a bacterium senses an attractant stimulus, the phosphorylation of CheY decreases due to the inhibited kinase activity of CheA and the dephosphorylation of CheY by CheZ [44]. Flagella begin to rotate counterclockwise and a swimming motion is achieved which pushes the cell forward [44]. All of the aforementioned Che proteins are present in the genome of *Y. pseudotuberculosis* [12]. Based on the established role of CheA/CheY in other bacteria, mutation in *cheA* or *cheY* was expected to hamper motility of *Y. pseudotuberculosis*, which was further confirmed by motility tests. At 3°C and 22°C, only the wild type strain was motile (Figures 3A and B), whereas none of the strains was motile at 28°C or 37°C (Figures 4A and B). The lack of motility of the IP32953 wild type strain at 28°C is in agreement with a previous study demonstrating impaired motility for this strain at 28°C [22].

Chemotaxis proteins belong to the flagellar regulon [45], and the expression of flagellar genes in *Y. enterocolitica* W22703 is highest at 20°C [46]. The genes encoding chemotaxis proteins are induced by an alternative sigma factor FliA and are repressed by an anti-sigma factor FlgM through the inactivation of FliA [45], [47], [48], both of which are regulated by the transcriptional activators FlhD and FlhC [45], [49]–[51] in *Y. enterocolitica* and *Y. pseudotuberculosis*. *Y. enterocolitica* and *Y. pseudotuberculosis* require FliA, FlhD, and FlhC for motility [49]–[52]. In *Y. enterocolitica*, *fliA* and *flgM* are transcribed at 25°C, but not at 37°C [47]. In addition to positively regulating flagellar genes, FliA negatively regulates several plasmid-encoded virulence genes at 25°C through VirF in *Y. enterocolitica* [53]. The observed increase in *cheA* and *cheY* expression at 3°C suggests that FliA may be present in *Y. pseudotuberculosis* at low temperature. Consequently, *Y. pseudotuberculosis* is motile at 3°C as shown in our study. Moreover, whether also virulence is down-regulated at 3°C, needs to be confirmed by further studies. Based on the aforementioned, we suggest that (1) the CheA/CheY two-component system is part of the low temperature sensing network of *Y. pseudotuberculosis*, (2) the CheA/CheY is needed for motility, and (3) at least an intact CheA is required for growth of *Y. pseudotuberculosis* at low temperature. Future research is warranted to investigate the role of the CheA/CheY two-component system in the regulation of the flagellation cascade.

An orphan response regulator, *YPTB1603*, predicted to encode a GerR family transcriptional regulatory protein [27], saw the third highest relative expression level at 3°C relative to the expression level at 28°C. The function of YPTB1603 in *Yersinia* has not previously been reported. A homolog of *YPTB1603* is *ECs0418* encoding a response regulator specific to *E. coli* O157:H7 strain Sakai [54]. The role of ECs0418 has not been studied. In addition to the response regulator receiver domain, an InterProScan search [55] suggested both the *YPTB1603* and the *ECs0418* contain a transcriptional regulator domain typical to LuxR family regulators that have been associated with several cellular and signaling events, including quorum sensing. It is thus intriguing to speculate that quorum sensing-type signals may be part of the low temperature sensing network. Our results demonstrate that the transcription of *YPTB1603* is enhanced at low temperature.

The genes that encode two-component systems KdpD/KdpE, YfhK/YfhA, PmrB/PmrA, NarX/NarP, CreC/CreB, RstB/RstA, BaeS/BaeR, SsrA/SsrB, HydH/HydG, EvgS/EvgA, PhoR/PhoB, EnvZ/OmpR, YPTB2728/YPTB2729, BarA/UvrY, YPTB2718/YPTB2719 and a multistep phosphorelay RcsC/YojN/RcsB had significantly higher (p-value<0.05) relative expression levels (median 4.0-fold) at 3°C than at 28°C (Table S3). Some of these signal transduction systems have been associated with survival from stresses posed by bile salts, low pH, hydrogen peroxide or increased osmolarity in previous studies (Table S5), but this study is the first to demonstrate the increased expression of these genes in cold stress in *Y. pseudotuberculosis*.

In the six two-component systems YehU/YehT, CpxA/CpxR, PhoQ/PhoP, CopS/CopR, NtrB/NtrC and ArcB/ArcA, the expression of only one of the two genes that encode a system differed with temperature. The results could be attributed to inactivation of certain components and re-established cross-talk between non-cognate kinase-regulator pairs. In *E. coli*, 7 of the 21 histidine kinases investigated can phosphorylate non-cognate response regulators in addition to the cognate regulators, and 9 of the 34 response regulators can be phosphorylated by non-cognate histidine kinases in addition to the cognate ones *in vitro* [56]. Cross-talk, however, is considered rare *in vivo* [10], [57]. Useful cross-talk, or cross-regulation, is seldom used when it is beneficial to combine multiple stimuli into one response or to expand a single stimulus to many responses [10].

The relative expression level of the *arcA* gene, which encodes the response regulator of the ArcB/ArcA two-component system was significantly higher (2.3; p-value<0.05), and that of *YPTB2099*, predicted to encode the orphan response regulator RssB, was significantly lower (0.2; p-value<0.05) at 3°C than at 28°C (Table S3). ArcA mainly regulates the transcription of genes involved in respiratory metabolism under anaerobic growth conditions in *E. coli* [58]. The histidine kinase ArcB phosphorylates both ArcA and RssB [59]. In log-phase cells or under low oxygen tension, the stationary-phase sigma factor σ^S^ is down-regulated because phosphorylated ArcA represses the transcription of *rpoS* which encodes σ^S^, and phosphorylated RssB targets σ^S^ for proteolysis by an ATP-driven ClpXP protease in *E. coli* [59], [60]. In the stationary phase or under high oxygen tension, the phosphorylation of ArcB diminishes, leading to the diminished phosphorylation of ArcA and RssB. Consequently, *rpoS* transcription increases, and σ^S^ proteolysis is reduced, resulting in a greater amount of σ^S^ [59]. A greater amount of σ^S^ enhances the expression of ArcA, leading to additional σ^S^ stabilization due to the competition of ArcA and RssB for phosphorylation by ArcB [59]. In *E. coli*, σ^S^ is induced at low temperature [1], as is the expression of *arcA* in *Y. enterocolitica* [40]. There are no studies on the role of σ^S^ and its regulation at low temperature in *Y. pseudotuberculosis*. However, our results indicate that the behavior of ArcA and RssB in *Y. pseudotuberculosis* could be similar to that in *E. coli*.

The results demonstrate that several signal transduction system encoding genes are upregulated at low temperature compared to optimum growth temperature in *Y. pseudotuberculosis*. The importance of the CheA/CheY two-component system in the growth of *Y. enterocolitica* at low temperatures was demonstrated by the impairment of growth of the *cheA* mutants at 3°C. The function of signal transduction systems at cold temperatures is probably arranged in complex networks with other cellular components. The identity of and interplay between these components is an important question and warrants further investigation. *Y. pseudotuberculosis*, a psychrotrophic pathogen with a wide temperature range for growth, requires mechanisms that facilitate its growth at low temperature. Long storage at cold favors the proliferation of *Y. pseudotuberculosis* over other bacteria in foods and has led to *Y. pseudotuberculosis* outbreaks through vegetables stored at low temperature. Investigation of the cold tolerance mechanisms may provide keys to novel strategies to control this psychrotrophic food pathogen in refrigerated foods.

## Supporting Information

Table S1 **Primers used in quantitative real-time reverse transcription-PCR in this study.**
(DOC)

Table S2 **C_q_ (quantification cycle) values and reaction efficiencies (E) of 16S rRNA gene**.
(DOC)

Table S3 **C_q_ (quantification cycle) values, reaction efficiencies (E), expression ratios (R), and standard deviations (SD) of Rs of the signal transduction system encoding genes of** ***Yersinia pseudotuberculosis*** **IP32953.**
(DOC)

Table S4 **Primers used in mutant construction and confirmation in this study.**
(DOC)

Table S5 **Predicted signal transduction systems of** ***Yersinia pseudotuberculosis*** **IP32953 and their known functions in** ***Yersinia*** **.**
(DOC)
