# Supplementary material for: Expression of Signal Transduction System Encoding Genes of Yersinia pseudotuberculosis IP32953 at 28°C and 3°C
Source: PLoS One. 2011 Sep 20;6(9):e25063. doi: 10.1371/journal.pone.0025063 (PMC3176822; doi:10.1371/journal.pone.0025063)
Supplement: Table S1 — Primers used in quantitative real-time reverse transcription-PCR in this study. (DOC) [file pone.0025063.s001.doc]

**Table S1.** Primers used in quantitative real-time reverse transcription-PCR in this study.

| Gene | Forward primer (5’→3’) | Reverse primer (5’→3’) |
| --- | --- | --- |
| 16S rRNA gene | GCTCGTGTTGTGAAATGTTGG | TATGTGGTCCGCTGGCTCT |
| *ntrB* | TCGTGTTGATGCGAGAGA | GTGCGGTCAGCGATAAAA |
| *ntrC* | CGTTTGGCTCGCTACTTTTT | CGCTTCGGTCTCTGGATG |
| *cpxA* | ATGGTCGGTCCCTTTTCC | ACATCATCAGCCGCATTTTT |
| *cpxR* | TGGAGTGAACAGCAACAAAA | AGCAGATAGAGCAGGGTGAA |
| *envZ* | CGTGAGATTTACCGTGAGTTGG | GCGACAGCCAGGTTTTGAG |
| *ompR* | ACGGAGCAAGGTTTTCAGGT | CCAACGATACGGTCCACTTCT |
| *arcB* | CACGGAAGAAGCACACAAAA | GCCCACTCACGCAATACC |
| *arcA* | TGAGCAGTGTAGGCGAAGAA | TGCGTGGCAGTTTGTAATG |
| *creC* | CGTTGGAATGATGTCTGGTTG | ATCTTGCGTTCACTGCGTTT |
| *creB* | AGCCCGTAGCGAAGAGTTG | AACCGCCAATAAGTGTGATTTT |
| *phoQ* | GCGACCTCTCGTTTACCTC | TGTTCACGCTCACCTTTTTC |
| *phoP* | GCCCTTCCAGATTGACCTTT | ACGACTTTCCCTGCGTTG |
| *cheA* | GGCGAAAGCACAGTCTCAA | ACATCCATCCCGACACCAC |
| *cheY* | AATGTGGAAGAAGCCGAAGA | TCACCATCAGAACAGGCAAC |
| *barA* | CGCAGTTATTCCAGGCTTTC | TTACCGCCCATTTCTTTCAC |
| *uvrY* | CAGCGTTCTTCTTGTTGATG | GCGACTTTGATACCTTTGATG |
| *rstB* | GGAATAAAACCATCGCAACA | CCAACCGCCAGAACATAG |
| *rstA* | ACCCTGTGCCGTGACCTAC | GCTGGCGGTGTCGTTTTT |
| *kdpD* | TTCGGTGTGATGCTGATTGT | GCCTGAAAACTGCTGGAAAG |
| *kdpE* | TGGCGAGTATTTGAGAGTGAG | CTTCATTGTTACGGGCAGA |
| *baeS* | GCCCACCTGCCTGAACAC | CAAATCGCCAACCCCAAC |
| *baeR* | AATCTACGGCGGAAACTG | ACAACAAACGGCACATCTCT |
| *yfhK* | GGCACAGCGTATCATTTGG | ATCGCCACCACCTCTTGT |
| *yfhA* | AAGCGATGGCAAAGAATGAA | GCCGACTCCCGTAATAGATG |
| *phoR* | CTGCCTGATGCCGTAGTT | TATGCTGCCCATTATCCTCT |
| *phoB* | CCGTGGCGAAGAAGAAGA | CCTGTGAGATGAAGGGTCAAG |
| *rcsC* | ATGTGGCGATTTGTGTGTTG | TTTGATTGACTGGCTTGCTCT |
| *yojN* | CAGCAAGCAAGGCGTGAG | GCAAATCGGTAGTGTCCTGTAATG |
| *rcsB* | CCGCATTACAAAAAGGGAAG | TTCAGCAAAGAGTCGCAAAA |
| *uhpB* | CTGGGTGATGTCCTGTTGTG | CCATTCGCTCTCATTTTGCT |
| *uhpA* | CTTCAGGGATGGGGGTCA | GCCTTCAGCCAACAGCAC |
| *pmrB* | CCTTTCCCCCTTGGTTGTT | GGCGTATTCCTGCGAGTG |
| *pmrA* | ATCAGGGGCAGAGTGACAAC | AGACGTGAGAGGATCGCAAA |
| *hydH* | CGCAATAATACCCATCAGTCC | GCATCGCATCTAACGCTT |
| *hydG* | AAAACTCCTGCGTGCTATCC | CTTCGCTTCGCTCTCTCAAC |
| *copS* | GGACTCGGGCTCTCCATC | TCTGGCTACTGGCAAACACA |
| *copR* | GTATCCAGGCGTTGTTGAGG | ACAGGGCGAACTCTTTGG |
| *evgS* | GGAGAAGGTGTTCGCAGTTT | GATTGGGCTATTGAGGAGGAG |
| *evgA* | GCAGTGAAGCATTGGAAAAA | GCAACGAGAAACCATCAAAA |
| *yehU* | ACAGATTTTGGCGGGAGA | CATCGTGGCTGCGTTTTAG |
| *yehT* | TTTGGCGAAGACACTGACAC | CTGAACAGGGGATATGACGAA |
| *narX* | ACCAAACCATCAAGCCATTC | TGCCCTATCCCACAACCA |
| *narP* | CAAAGATTGCGAGCCAGAA | ACATCCCACGAGCCACCT |
| *YPTB2728* | CTACCAAAGCGGCAGTGAA | AGCCCACCCCAGAGATAAAG |
| *YPTB2729* | GCAAACGCCCATTATTTTTC | AGTCGTCAGCACCCAACTCT |
| *YPTB2718* | TGATAAACATCGCCGTGAAA | TAAATCCCCCTGTGCTACCC |
| *YPTB2719* | ATGCCTAAACCGTGCTATCC | TTACCCTGCCATTCGCTAC |
| *YPTB0311* | TCC CTTTCGCTCTGGTTATTT | CGCTCCTCCACTTTCATTTCT |
| *YPTB0310* | GAAGAAAAGTCCCCAGCAAA | AGAAAGTTGAGCAGCAATAACC |
| *YPTB3808* | ACCCCGCCTCTCCTTCTT | CACCGCTACGCCACTCAC |
| *YPTB3801* | CTGTGAAGTGCTGCGAGATG | TAAGTGCGATTGAGGGCTGT |
| *YPTB2099* | GGATTGAGTTTGTTGAGCAGTT | TCCGCCATCTTGTCAGTT |
| *YPTB3350* | TGGCAATGTTCGTGAATTAGAG | GGCTGAATCGTTGAAAAAGG |
| *YPTB1603* | TGGTCAAAGGGATAAGTGTAAAAG | GCTGATGCTGTAGTGCGAAG |
| *ibpA* | TCTTGCTCCATTGTATCGTTC | ATGCGGTAGTTATTTTCGTCA |
