# Supplementary material for: Expression of Signal Transduction System Encoding Genes of Yersinia pseudotuberculosis IP32953 at 28°C and 3°C
Source: PLoS One. 2011 Sep 20;6(9):e25063. doi: 10.1371/journal.pone.0025063 (PMC3176822; doi:10.1371/journal.pone.0025063)
Supplement: Table S2 — Cq (quantification cycle) values and reaction efficiencies (E) of 16S rRNA gene. (DOC) [file pone.0025063.s002.doc]

**Table S2.** C_q_ (quantification cycle) values and reaction efficiencies (E) of 16S rRNA gene*.*

| Dilution series | Sample^a^ | $\overline{x}(\text{C}_{\text{q},28^{\circ}\text{C}})$^b^ | $\overline{x}(\text{C}_{\text{q},3^{\circ}\text{C}})$^b^ | ΔC_q_^c^ | E |
| --- | --- | --- | --- | --- | --- |
| I | Ia | 15.83 | 15.17 | 0.66 | 0.99 |
|  | Ib | 15.74 | 15.39 | 0.35 |  |
|  | IIa | 15.82 | 14.88 | 0.94 |  |
|  | IIb | 15.92 | 14.82 | 1.1 |  |
|  | IIIa | 15.86 | 14.98 | 0.88 |  |
|  | IIIb | 15.65 | 15.29 | 0.36 |  |
| II | Ia | 16.14 | 15.46 | 0.68 | 1.03 |
|  | Ib | 15.94 | 14.96 | 0.98 |  |
|  | IIa | 16.31 | 15.09 | 1.22 |  |
|  | IIb | 16.22 | 14.99 | 1.23 |  |
|  | IIIa | 15.99 | 15.01 | 0.98 |  |
|  | IIIb | 16.02 | 14.87 | 1.15 |  |
| III | Ia | 16.81 | 16.18 | 0.63 | 1.03 |
|  | Ib | 16.66 | 15.90 | 0.76 |  |
|  | IIa | 16.73 | 16.04 | 0.69 |  |
|  | IIb | 16.68 | 15.96 | 0.72 |  |
|  | IIIa | 16.56 | 16.03 | 0.53 |  |
|  | IIIb | 16.51 | 15.82 | 0.69 |  |
| IV | Ia | 16.69 | 15.65 | 1.04 | 1.06 |
|  | Ib | 16.33 | 15.51 | 0.82 |  |
|  | IIa | 16.47 | 15.44 | 1.03 |  |
|  | IIb | 16.26 | 15.48 | 0.78 |  |
|  | IIIa | 16.25 | 15.42 | 0.83 |  |
|  | IIIb | 16.19 | 15.32 | 0.87 |  |

^a^I, II, III represent biological replicates, a and b represent parallel reverse transcription reactions

^b^Average of three PCR replicates

^c^ΔC_q_= $\overline{x}(\text{C}_{\text{q},28^{\circ}\text{C}})-\overline{x}(\text{C}_{\text{q},3^{\circ}\text{C}})$
