# Supplementary material for: Expression of Signal Transduction System Encoding Genes of Yersinia pseudotuberculosis IP32953 at 28°C and 3°C
Source: PLoS One. 2011 Sep 20;6(9):e25063. doi: 10.1371/journal.pone.0025063 (PMC3176822; doi:10.1371/journal.pone.0025063)
Supplement: Table S3 — Cq (quantification cycle) values, reaction efficiencies (E), expression ratios (R), and standard deviations (SD) of Rs of the signal transduction system encoding genes of Yersinia pseudotuberculosis IP32953. (DOC) [file pone.0025063.s003.doc]

**Table S3.** C_q_ (quantification cycle) values, reaction efficiencies (E), expression ratios (R), and standard deviations (SD) of Rs of the signal transduction system encoding genes of *Yersinia pseudotuberculosis* IP32953.

| Gene | $\overline{x}(\text{C}_{\text{q},28^{\circ}\text{C}})^{\text{a}}$ | $\overline{x}(\text{C}_{\text{q},3^{\circ}\text{C}})^{\text{a}}$ | ΔC_q_^b^ | E | $\overline{x}(\text{R})^{\text{c}}$ | SD(R) | p-value |
| --- | --- | --- | --- | --- | --- | --- | --- |
| Significantly (p-value < 0.05) different expression between 3°C and 28°C | | | | | | | |
| *cheA* | 22.17 | 16.36 | 5.81 | 0.95 | 30.6 | 8.8 | 0.002 |
| *cheY* | 20.91 | 15.31 | 5.60 | 0.93 | 24.8 | 5.6 | 0.002 |
| *YPTB1603* | 24.66 | 20.05 | 4.61 | 0.98 | 12.7 | 3.8 | 0.006 |
| *kdpD* | 26.92 | 23.29 | 3.63 | 1.19 | 9.0 | 1.2 | 0.001 |
| *yfhK* | 24.09 | 20.40 | 3.69 | 1.09 | 7.5 | 2.0 | 0.007 |
| *pmrB* | 22.05 | 18.61 | 3.44 | 1.01 | 5.4 | 1.1 | 0.005 |
| *narP* | 19.34 | 16.28 | 3.06 | 1.01 | 5.4 | 1.0 | 0.005 |
| *yehU* | 26.31 | 23.36 | 2.95 | 1.04 | 5.2 | 1.4 | 0.011 |
| *creC* | 21.44 | 18.20 | 3.24 | 0.93 | 5.2 | 0.8 | 0.001 |
| *rstB* | 22.03 | 18.85 | 3.18 | 0.94 | 5.1 | 1.2 | 0.005 |
| *rcsB* | 17.84 | 14.73 | 3.11 | 0.96 | 5.1 | 0.8 | 0.002 |
| *cpxA* | 21.56 | 18.58 | 2.98 | 0.99 | 5.0 | 1.3 | 0.008 |
| *baeS* | 24.67 | 21.81 | 2.85 | 1.05 | 4.9 | 1.1 | 0.007 |
| *narX* | 25.43 | 22.35 | 3.08 | 0.92 | 4.8 | 1.1 | 0.008 |
| *YPTB0311* | 23.28 | 20.25 | 3.03 | 1.06 | 4.7 | 0.6 | 0.001 |
| *hydH* | 27.57 | 24.45 | 3.13 | 0.98 | 4.5 | 1.2 | 0.01 |
| *hydG* | 27.30 | 24.28 | 3.02 | 1.08 | 4.4 | 0.6 | 0.002 |
| *evgA* | 21.27 | 18.11 | 3.17 | 1.01 | 4.4 | 0.5 | 0.001 |
| *phoQ* | 18.74 | 15.89 | 2.85 | 0.97 | 4.4 | 0.8 | 0.005 |
| *yfhA* | 22.44 | 19.36 | 3.08 | 0.96 | 4.3 | 1.1 | 0.014 |
| *phoB* | 19.20 | 16.12 | 3.08 | 1.01 | 4.2 | 0.7 | 0.003 |
| *evgS* | 20.60 | 17.57 | 3.03 | 1.02 | 4.1 | 0.5 | 0.002 |
| *YPTB0310* | 23.54 | 20.43 | 3.11 | 0.92 | 4.0 | 0.4 | 0.001 |
| *YPTB3801* | 20.84 | 18.04 | 2.80 | 1.01 | 3.9 | 1.6 | 0.034 |
| *envZ* | 21.02 | 18.59 | 2.43 | 1.06 | 3.6 | 0.9 | 0.013 |
| *rcsC* | 20.96 | 18.23 | 2.74 | 1.04 | 3.4 | 0.7 | 0.014 |
| *YPTB2729* | 20.63 | 18.33 | 2.30 | 1 | 3.2 | 1.0 | 0.028 |
| *yojN* | 19.93 | 17.37 | 2.56 | 1.02 | 2.9 | 0.6 | 0.005 |
| *copR* | 24.30 | 22.21 | 2.09 | 1.08 | 2.9 | 0.7 | 0.018 |
| *rstA* | 22.26 | 20.18 | 2.09 | 1.08 | 2.9 | 0.6 | 0.001 |
| *phoR* | 18.53 | 16.02 | 2.51 | 0.98 | 2.7 | 0.8 | 0.024 |
| *kdpE* | 19.94 | 17.72 | 2.22 | 0.93 | 2.7 | 0.7 | 0.007 |
| *creB* | 19.58 | 17.49 | 2.10 | 0.94 | 2.5 | 0.4 | 0.009 |
| *uvrY* | 21.95 | 19.93 | 2.03 | 0.97 | 2.4 | 0.3 | 0.007 |
| *ntrB* | 19.34 | 17.26 | 2.08 | 0.93 | 2.4 | 0.4 | 0.008 |
| *baeR* | 19.53 | 17.20 | 2.33 | 0.99 | 2.4 | 0.3 | 0.004 |
| *YPTB2728* | 24.22 | 22.00 | 2.22 | 0.97 | 2.4 | 0.4 | 0.013 |
| *barA* | 22.18 | 20.06 | 2.12 | 0.86 | 2.4 | 0.8 | 0.048 |
| *YPTB2718* | 21.37 | 19.27 | 2.10 | 0.88 | 2.4 | 0.4 | 0.013 |
| *arcA* | 18.94 | 17.05 | 1.89 | 0.99 | 2.3 | 0.4 | 0.003 |
| *ompR* | 19.26 | 17.43 | 1.83 | 1 | 2.2 | 0.5 | 0.029 |
| *YPTB3350* | 30.11 | 28.14 | 1.96 | 0.99 | 2.1 | 0.5 | 0.045 |
| *pmrA* | 20.28 | 18.16 | 2.13 | 0.92 | 1.9 | 0.2 | 0.012 |
| *YPTB2719* | 19.69 | 18.08 | 1.60 | 0.91 | 1.8 | 0.5 | 0.047 |
| *YPTB2099* | 22.58 | 23.93 | -1.34 | 1.11 | 0.2 | 0.0 | 0.004 |
| Non-significantly (p-value ≥ 0.05) different expression between 3°C and 28°C | | | | | | | |
| *uhpB* | 22.25 | 20.36 | 1.89 | 1.06 | 2.0 | 0.7 | 0.113 |
| *copS* | 25.11 | 23.41 | 1.70 | 1.01 | 1.8 | 0.4 | 0.067 |
| *ntrC* | 22.99 | 21.41 | 1.58 | 0.9 | 1.7 | 0.5 | 0.091 |
| *cpxR* | 22.36 | 21.18 | 1.18 | 1.04 | 1.5 | 0.5 | 0.241 |
| *phoP* | 16.48 | 15.32 | 1.16 | 0.93 | 1.3 | 0.2 | 0.062 |
| *uhpA* | 20.63 | 19.51 | 1.13 | 1.04 | 1.1 | 0.4 | 0.754 |
| *YPTB3808* | 24.11 | 23.23 | 0.88 | 0.97 | 1.0 | 0.4 | 0.867 |
| *arcB* | 22.59 | 21.91 | 0.68 | 1.04 | 1.0 | 0.3 | 0.92 |
| *yehT* | 21.16 | 20.91 | 0.25 | 1.04 | 0.6 | 0.2 | 0.088 |

^a^Average of the RT, PCR, and biological replicates

${}^{\text{b}}\Delta\text{C}_{\text{q}}=\overline{x}(\text{C}_{\text{q},28^{\circ}\text{C}})-\overline{x}(\text{C}_{\text{q},3^{\circ}\text{C}})$

$${}^{\text{c}}\text{R}=\frac{\left(1+\text{E}_{\text{gene}}\right)^{\Delta\text{C}_{\text{q,gene}}}}{\left(1+\text{E}_{16\text{SrRNAgene}}\right)^{\Delta\text{C}_{\text{q},16\text{SrRNAgene}}}}$$
