# Supplementary material for: Expression of Signal Transduction System Encoding Genes of Yersinia pseudotuberculosis IP32953 at 28°C and 3°C
Source: PLoS One. 2011 Sep 20;6(9):e25063. doi: 10.1371/journal.pone.0025063 (PMC3176822; doi:10.1371/journal.pone.0025063)
Supplement: Table S4 — Primers used in mutant construction and confirmation in this study. (DOC) [file pone.0025063.s004.doc]

**Table S4. Primers used in mutant construction and confirmation in this study.**

| Primer | Sequence (5’→3’) |
| --- | --- |
| *cheA*30-IBS | AAAAAAGCTTATAATTATCCTTAGCGTTCTATCAGGTGCGCCCAGATAGGGTG |
| *cheA*30-EBS1d | CAGATTGTACAAATGTGGTGATAACAGATAAGTCTATCAGACTAACTTACCTTTCTTTGT |
| *cheA*30-EBS2 | TGAACGCAAGTTTCTAATTTCGATTAACGCTCGATAGAGGAAAGTGTCT |
| EBS Universal | CGAAATTAGAAACTTGCGTTCAGTAAAC |
| T7 | TAATACGACTCACTATAGGG |
| *cheA30*-flank-left | GCATGGATATTACCGCGTTT |
| *cheA30*-flank-right | GAGCACCATCCAACAGGTTT |
| *cheA243*-IBS | AAAAAAGCTTATAATTATCCTTAAGTGCCCAAGCGGTGCGCCCAGATAGGGTG |
| *cheA243*-EBS1d | CAGATTGTACAAATGTGGTGATAACAGATAAGTCCAAGCGCATAACTTACCTTTCTTTGT |
| *cheA243*-EBS2 | TGAACGCAAGTTTCTAATTTCGATTGCACTTCGATAGAGGAAAGTGTCT |
| *cheY243*-IBS | AAAAAAGCTTATAATTATCCTTATTGGCCACGTTGGTGCGCCCAGATAGGGTG |
| *cheY243*-EBS1d | CAGATTGTACAAATGTGGTGATAACAGATAAGTCACGTTGCCTAACTTACCTTTCTTTGT |
| *cheY243*-EBS2 | TGAACGCAAGTTTCTAATTTCGGTTGCCAATCGATAGAGGAAAGTGTCT |
| *cheA243*-flank-left | AAACCTGTTGGATGGTGCTC |
| *cheA243*-flank-right | TCGGTAGCGGATTGATTTTC |
| *cheY243*-flank-left | GCAGGTGGTTTCGATTTTGT |
| *cheY243*-flank-right | TGCTGCGATGATGTTCTCTT |
| N*inv-*left | TAAGGGTACTATCGCGGCGGA |
| N*inv*-right | CGTGAAATTAACCGTCACACT |
| K*virF-*left | TCGTGGCAGCTATGCTGTTC |
| K*virF-*right | ATACGTCGCTCGCTTATCCA |
| probe-left | TGGCAATGATAGCGAAACAA |
| probe-right | GGTACCGCCTTGTTCACATT |
