# Supplementary material for: Expression of Signal Transduction System Encoding Genes of Yersinia pseudotuberculosis IP32953 at 28°C and 3°C
Source: PLoS One. 2011 Sep 20;6(9):e25063. doi: 10.1371/journal.pone.0025063 (PMC3176822; doi:10.1371/journal.pone.0025063)
Supplement: Table S5 — Predicted signal transduction systems of Yersinia pseudotuberculosis IP32953 and their known functions in Yersinia . (DOC) [file pone.0025063.s005.doc]

**Table S5.** Predicted signal transduction systems of *Yersinia pseudotuberculosis* IP32953 and their known functions in *Yersinia*.

| Signal transduction system^a^ | Locus tag in IP32953 | Species | Function |
| --- | --- | --- | --- |
| Complete signal transduction systems | | | |
| NtrB (HK) | *YPTB0023* | *Y. pseudotuberculosis* | Resistance to bile salts [26], *ntrB* induced at 3°C (this study) |
| NtrC (RR) | *YPTB0022* |  |  |
| CpxA (HK) | *YPTB0069* | *Y. pseudotuberculosis* | Contact with eukaryotic cells, type III secretion [18,19], *cpxA* induced at 3°C (this study) |
| CpxR (RR) | *YPTB0070* |  |  |
|  |  | *Y. enterocolitica* | Extracytoplasmic stress response, *cpxA* indispensable for growth [61-63] |
|  |  | *Y. pestis* | Resistance to neutrophils [64] |
| SsrA (HK, RR) | *YPTB0311* | *Y. pseudotuberculosis* | Genes induced at 3°C (this study) |
| SsrB (RR) | *YPTB0310* |  |  |
|  |  | *Y. enterocolitica* | Induction of virulence factor SrfA [40], *ssrB* induced at low temperature [42] |
| PmrB (HK) | *YPTB0468* | *Y. pseudotuberculosis* | Resistance to acids and bile salts [26], possibly involved in peptidoglycan homeostasis [14], genes induced at 3°C (this study) |
| PmrA (RR) | *YPTB0469* |  |  |
|  |  | *Y. pestis* | Resistance to antimicrobials [65] |
| ArcB (HK, Hpt) | *YPTB3500* | *Y. pseudotuberculosis* | Resistance to hydrogen peroxide and bile salts [26], *arcA* induced at 3°C (this study) |
| ArcA (RR) | *YPTB0601* |  |  |
|  |  | *Y. enterocolitica* | *arcA* induced at low temperature [40] |
| BarA (HK, RR, Hpt) | *YPTB0750* | *Y. pseudotuberculosis* | Mediation on the expression of virulence genes [20] , genes induced at 3°C (this study) |
| UvrY (RR) | *YPTB1735* |  |  |
|  |  | *Y. ruckeri* | Invasion of epithelial cells, resistance to hydrogen peroxide [66,67] |
| YehU (HK) | *YPTB3788* | *Y. pseudotuberculosis* | *yehU* induced at 3°C (this study) |
| YehT (RR) | *YPTB0840* |  |  |
| PhoR (HK) | *YPTB0918* | *Y. pseudotuberculosis* | Genes induced at 3°C (this study) |
| PhoB (RR) | *YPTB0917* |  |  |
| HydH (HK) | *YPTB1204* | *Y. pseudotuberculosis* | Genes induced at 3°C (this study) |
| HydG (RR) | *YPTB1205* |  |  |
| RcsC (HK, RR) | *YPTB1257* | *Y. pseudotuberculosis* | Cell envelope, virulence, motility, regulation of biofilm formation, susceptibility to bile salts [22,26,68], genes induced at 3°C (*Y. pseudotuberculosis*, this study) |
| YojN (HPt) | *YPTB1259* | *Y. enterocolitica* |  |
| RcsB (RR) | *YPTB1258* |  |  |
|  |  | *Y. pestis* | Regulation of biofilm formation [23,69-71] |
| EvgS (HK) | *YPTB1922* | *Y. pseudotuberculosis* | Genes induced at 3°C (this study) |
| EvgA (RR) | *YPTB1923* |  |  |
| NarX (HK) | *YPTB1957* | *Y. pseudotuberculosis* | Genes induced at 3°C (this study) |
| NarP (RR) | *YPTB2763* |  |  |
| CopS (HK) | *YPTB1992* | *Y. pseudotuberculosis* | *copR* induced at 3°C (this study) |
| CopR (RR) | *YPTB1993* |  |  |
| RstB (HK) | *YPTB2231* | *Y. pseudotuberculosis* | Virulence, resistance to hydrogen peroxide [26], genes induced at 3°C (this study) |
| RstA (RR) | *YPTB2230* |  |  |
| CheA (HK) | *YPTB2405* | *Y. pseudotuberculosis* | Cold tolerance (this study) |
| CheY (RR) | *YPTB2397* |  |  |
|  |  | *Y. enterocolitica* | *cheA* [40] and *cheY* [42] induced at low temperature |
|  |  | *Y. pestis* | CheY involved in resistance to neutrophils [64] |
| PhoQ (HK) | *YPTB2435* | *Y. pseudotuberculosis* | Virulence and survival in macrophages, resistance to antimicrobials, low pH and osmotic stress; regulation of biofilms; susceptibility to hydrogen peroxide and bile salts [13-17,26], *phoQ* induced at 3°C (this study) |
| PhoP (RR) | *YPTB2434* |  |  |
|  |  | *Y. enterocolitica* | *phoP* induced at low temperature [42] |
|  |  | *Y. pestis* | Virulence, and survival in macrophages and neutrophils; Mg^2+^ homeostasis; resistance to low pH, oxidative stress, high osmolarity and antimicrobials; regulation of metabolic processes, regulation of biofilms [16,64,65,71-76] |
| YPTB2718 (HK) | *YPTB2718* | *Y. pseudotuberculosis* | Genes induced at 3°C (this study) |
| YPTB2719 (RR) | *YPTB2719* |  |  |
| YPTB2728 (HK) | *YPTB2728* | *Y. pseudotuberculosis* | Genes induced at 3°C (this study) |
| YPTB2729 (RR) | *YPTB2729* |  |  |
| BaeS (HK) | *YPTB2817* | *Y. pseudotuberculosis* | Function in type III secretion investigated, not found [18], genes induced at 3°C (this study) |
| BaeR (RR) | *YPTB2818* |  |  |
| YfhK (HK) | *YPTB2876* | *Y. pseudotuberculosis* | Involved in the synthesis of glucosamine-6-phosphate, virulence and antimicrobial agent susceptibility [25,26], genes induced at 3°C (this study) |
| YfhA (RR) | *YPTB2874* |  |  |
| KdpD (HK) | *YPTB2919* | *Y. pseudotuberculosis* | Genes induced at 3°C (this study) |
| KdpE (RR) | *YPTB2920* |  |  |
|  |  | *Y. pestis* | Resistance to neutrophils [64] |
| CreC (HK) | *YPTB3170* | *Y. pseudotuberculosis* | Genes induced at 3°C (this study) |
| CreB (RR) | *YPTB3171* |  |  |
| EnvZ (HK) | *YPTB3763* | *Y. pseudotuberculosis* | Resistance to osmotic stress, low pH and bile salts; flagella biosynthesis, susceptibility to antimicrobials, virulence [23,24,26], genes induced at 3°C (this study) |
| OmpR (RR) | *YPTB3764* |  |  |
|  |  | *Y. enterocolitica* | Flagella biosynthesis; resistance to osmotic, low pH, oxidative and high temperature stresses, virulence, susceptibility to antimicrobials [77-82] |
| UhpB (HK) | *YPTB3846* | no *Yersinia* data | Unknown |
| UhpA (RR) | *YPTB3847* |  |  |
| Orphans |  |  |  |
| YPTB1603 | *YPTB1603* | *Y. pseudotuberculosis* | Gene induced at 3°C (this study) |
| YPTB2099 | *YPTB2099* | *Y. pseudotuberculosis* | Gene down-regulated at 3°C (this study) |
| YPTB3350 | *YPTB3350* | *Y. pseudotuberculosis* | Gene induced at 3°C (this study) |
| YPTB3801 | *YPTB3801* | *Y. pseudotuberculosis* | Gene induced at 3°C (this study) |
| CvgSY (HK, RR) | *YPTB3808* | *Y. pseudotuberculosis* | Virulence [21] |
|  |  | *Y. pestis* | Resistance to neutrophils [64] |

^a^HK, histidine kinase; RR, response regulator; Hpt, histidine phosphotransferase
